# A Regulatory Network Analysis of the Importance of USP15 in Breast Cancer Metastasis and Prognosis

**DOI:** 10.1155/2022/1427726

**Published:** 2022-09-29

**Authors:** Jun Ling, Chenhui Qin, Tao Li, Baozhen Wang, Weiji Cai, Lei Ma, Yanfeng Wang, Jing Chen, Fang Xu

**Affiliations:** ^1^School of Basic Medical Sciences, Ningxia Medical University, Yinchuan 750004, China; ^2^Key Laboratory of Fertility Preservation and Maintenance (Ningxia Medical University), Ministry of Education, Yinchuan 750004, China; ^3^School of Clinical Medicine, Ningxia Medical University, Ningxia, China; ^4^The General Hospital of Ningxia Medical University, Ningxia, China; ^5^Department of Surgical Oncology, Tumor Hospital, The General Hospital of Ningxia Medical University, Ningxia, China

## Abstract

**Background:**

Ubiquitin-specific protease15(USP15), is the 16th identified protease in the USP family and is a key protein in tumorigenesis. However, the predictive value and regulatory mechanism of USP15 in breast cancer are unclear.

**Methods:**

The GEPIA, UALCAN, GeneMANIA, and STRING databases were applied to explore the expression of USP15 in breast cancer and associated proteins. In addition, the TIMER database was evaluated for immune infiltration patterns. Moreover, protein immunoblotting assay, cell scratching assay, small compartment invasion assay, 3D stromal gel assay, immunoprecipitation assay, and immunohistochemistry (IHC) were used to USP15 regulatory mechanisms in breast cancer.

**Results:**

In BRCA, several databases, including GEPIA and UALCAN, describe the upregulation of total protein levels and USP15 phosphorylation. In addition, the expression of USP15 was significantly correlated with gender and clinical stage. Overall survival (OS) was lower in patients with high USP15 expression. Functional network analysis showed that USP15 is involved in tumor-associated pathways, DNA replication, and cell cycle signaling through TGF*β*RI. In addition, USP15 expression was positively correlated with immune infiltration, including immune score, mesenchymal score, and several tumor-infiltrating lymphocytes (TIL). In addition, IHC results further confirmed the high expression of USP15 in breast cancer and its prognostic potential.

**Conclusions:**

These findings demonstrate that high USP15 expression indicates poor prognosis in BRCA and reveal potential regulatory networks and the positive relationship with immune infiltration. Thus, USP15 may be an attractive predictor for BRCA.

## 1. Introduction

Breast cancer is the most important disease threatening the life and health of women worldwide [1–3]. Among women, breast cancer incidence and mortality have ranked first in the world for the past five years. Breast cancer is divided into four types based on the expression of Estrogen Receptor (ER), Progesterone Receptor (PR), Human Epidermal Growth Factor (HER2), and Ki-67: Luminal A, Luminal B, HER2-Enriched and Three Breast Cancer Negative (TNBC) [4]. Among them, TNBC is a type of breast cancer that is negative for the expression of ER, PR, and HER2 and has a high degree of metastasis and invasiveness. The clinical manifestations of this type of breast cancer are poor prognosis [5, 6]. With the development of clinical treatment, treatment methods for TNBC are constantly changing and improving, but the current clinical treatment effect is still not satisfactory [6–8]. Recent routine clinical surgeries, drug radiation therapy, and chemotherapy fail to improve the prognosis and survival of patients with TNBC. However, with the advent of targeted drug therapy technology bringing hope to patients with TNBC, we urgently need to find new tumor markers to advance the treatment of clinical breast cancer [8].

Transforming growth factor (TGF*β*) is a multifunctional cytokine that regulates the cell cycle and affects cell proliferation, differentiation, adhesion, and metastasis in tumor formation and development [9–11]. The Smad family is the first kinase substrate of the TGF*β* receptor whose involvement in the TGF*β* signaling pathway has been confirmed [12–14]. Activated TGF*β*, when stimulated by an external signal, first binds to the type II TGF*β* receptor to form a complex [15], which continues to recruit both type I receptors. Then, a complex is developed to phosphorylate further and activate its downstream signaling molecules smad2 and smad3. After phosphorylation and activation, smad2 and smad3 form a trimeric complex with smad4, enter the nucleus and regulate the transcription and expression of nuclear genes under the action of DNA molecules, recruit of [16]. The TGF*β* signaling pathway is vital in many tumors such as breast cancer, lung cancer, etc. [17].

Epigenetic changes influence tumor development, with protein ubiquitination, a common form of posttranslational modification, playing a crucial role in tumor formation, the reverse process of which is called deubiquitination [18, 19], which refers to the ability of already ubiquitinated proteins to separate ubiquitin and substrate protein molecules under the catalytic action of deubiquitinating enzymes [20]. The ubiquitin-specific proteases (USPs) are the largest family of deubiquitinases (DUBs). USPs can play an important role in many physiological activities of the human body via the ubiquitin-proteasome (UPP) pathway by affecting the expression of circulating signaling pathways such as transforming growth factor (TGF*β*) and p53. [21]. These include ubiquitin-specific protease 15(USP15), a key carcinogen protein highly expressed in skin cancer and blood-associated tumors. However, USP15 may also promote the formation and development of tumors by activating signaling pathways [22–24].

USP15 acts as a “biological thermostat” in the TGF-*β* pathway [25]. In normal tissue cells, the expression level of TGF*β* is balanced. When the level of TGF*β* expression in cells is too high, smad7 binds to smurf2 downstream of its signaling pathway to form a complex that further binds to TGF*β* type I receptors to facilitate ubiquitination and degradation type I TGF*β* receptors. The body is stable to induce receptors, thereby reducing the expression level of TGF*β* [26, 27]. On the contrary, when the level of TGF*β* in the body is too low, USP15 associates with smad7 and smurf2 to form a complex to deubiquitinate the type I TGF*β* receptor and further increase the expression level of the intracellular signaling of TGF*β* [28, 29]. However, when USP15 is overexpressed, the body perceives TGF*β* levels to be underexpressed and may tip the balance towards deubiquitination [25], leading to overexpression of TGF*β* and thus promoting tumor proliferation and migration [30, 31], The only commercially available small molecule compound with significant inhibitory effects on USP15 is the broad-spectrum ubiquitination inhibitor PR-619, which inevitably inhibits other deubiquitination enzymes while inhibiting USP15, causing several unknown side effects [32, 33]. Therefore, it becomes particularly important to study the predictive value and regulatory mechanisms of USP15 in breast cancer to guide breast cancer treatment [24].

## 2. Materials and Methods

### 2.1. UALCAN

UALCAN (http://ualcan.path.uab.edu/analysis.html), a comprehensive web resource, provides analyses based on The Cancer Genome Atlas (TCGA) and MET500 cohort data. In our study, expression data for USP15 was obtained using the “Expression Analysis” module of UALCAN Student's *t*-test was used to generate a *p* value. The *p* value cutoff was 0.05. Predictive analysis was performed using a Kaplan–Meier curve [34–36].

### 2.2. GEPIA

GEPIA (http://gepia.cancer-pku.cn/index.html) is an analysis tool containing RNA sequence expression data of 9736 tumors and 8587 normal tissue samples developed at Peking University. In this study, we performed with the “Multiple Gene Comparison” module of GEPIA. Predictive analysis was performed using a Kaplan–Meier curve. The *p* value cutoff was 0.05. The student's *t*-test was used to generate a *p* value for expression or pathological stage analysis [37, 38].

### 2.3. LinkedOmics

LinkedOmics (LinkedOmics) is a publicly available portal that includes multiomics data from all 32 TCGA Cancer types and 10 Clinical Proteomics Tumor Analysis Consortium (CPTAC) cancer cohorts. The web application has three analytical modules: LinkFinder, LinkInterpreter, and LinkCompare [38, 39].

### 2.4. String

STRING (https://string-db.org/) aims to collect, score, and integrate all publicly available sources of protein-protein interaction (PPI) data, and to complement these with computational predictions of potential functions. We conducted a PPI network analysis of differentially expressed USP15 to explore the interactions among them with STRING [36, 40].

### 2.5. GeneMANIA

GeneMANIA (http://www.genemania.org) is a user-friendly website that provides information on protein and genetic interactions, pathways, coexpression, colocalization, and protein domain similarity of submitted genes [37, 41].

### 2.6. TIMER

TIMER (https://cistrome.shinyapps.io/timer/) is a reliable, intuitive tool that systematically evaluates different immune cells' infiltration and their clinical impact. In our study, the “Gene module” was used to assess the correlation between USP15 and the infiltration of immune cells. “Survival module” was used to evaluate the correlation between clinical outcomes and the infiltration of immune cells and USP15 expression [36, 42].

### 2.7. Immunohistochemical (IHC) Staining

Breast cancer and normal tissue sections were provided by the General Hospital of Ningxia Medical University, China. The tissue section array included 20 cases of breast cancer and 35 cases of normal tissue. This tissue section was used for immunohistochemical staining. IHC staining was performed as previously described [43, 44].

### 2.8. Cell Culture

MDA-MB-231, BT549, T47D, SUM159, BT-20, MDA-MB-468 and MCF7 (breast cancer cells), MCF-10A **(**Human Mammary Epithelial Cells), and GES-1 (mucosal epithelial cells human gastric) from ATCC (Manasseh's, VA, USA) purchased or donated to China Normal University by Professor Liu Mingyao's research group. Cells were maintained in DMEM or Death Complete 1640 medium supplemented with 10% fetal bovine serum (FBS) and 1% penicillin/streptomycin. All cells were maintained at 37°C in a 5% CO_2_ incubator. All cell lines were regularly checked for mycoplasma contamination.

### 2.9. Chemicals

PR619 was purchased from Selleck's official website (https://www.selleck.cn/*No*.s7130). Compound stock solutions were prepared in DMSO at a concentration of 100 mM and stored at -20°C.

### 2.10. Transwell Invasion Assay

Transwell invasion assay was performed following the manufacturer's instructions with modifications. We selected 1^∗^10^5^ cells per well and cultured the FBS concentration at 10%. Breast cancer cells were resuspended in a medium with test compounds and seeded on transwell filters (8 *μ*m pore size; Millipore) precoated with Matrigel or Collagen I. After 12 h, cells on the top side of the filters were wiped by cotton swaps. Cells on the lower side were then fixed in 4% paraformaldehyde and stained with 0.1% crystal violet. Images were taken under an inverted microscope (Olympus) [45, 46].

### 2.11. Sulforhodamine B (Sulforhodamine B, SRB) Assay

Cells with good growth conditions were selected, digested with trypsin, centrifuged, resuspended, adjusted to a cell density of 3000 cells/mL, and inoculated into 96-well plates, 100 *μ*L per well, with 3 replicate wells and 5 for each group. Drug treatment was given after 24 hours of incubation in a 5% CO_2_ incubator. The cells were fixed with trichloroacetic acid (TCA) after the drug-addled culture (96 h). Place at 4°C for 1 hour, spin dry, and dry at room temperature. After drying, 50 *μ*L of 0.4% SRB solution was added to each well, and the cells were stained for 10 min at room temperature in the dark. Subsequent 5 washes removed unbound cell dye with 1% acetic acid. Leave the 96-well plate to dry at room temperature. 200*μ*L of 10 mM Trisbase (pH = 10.5) was added, and the plate was shaken until completely dissolved. The OD value of each well at a wavelength of 515 nm was measured on a microplate reader. IC_50_ values were calculated [47, 48].

### 2.12. 3D On-Top Culture

3D culture assay was conducted as described. Briefly, MDA-MB-231 breast cancer cells were seeded on a 48-well plate coated with a thin layer of Matrigel. Thirty minutes postseeding, a medium containing 10% Matrigel and different concentrations of PR619 were added to the plated culture. The culture was maintained for 4 days and the on-top Matrigel–medium mixture with or without PR619 was replaced every 2 days. 3D on-top culture was performed as previously described [45, 49].

### 2.13. Immunoblotting Analysis

Cells were lysed in RIPA buffer (50 mM Tris pH 8.0, 150 mM NaCl, 1% NP-40, 0.5 mM EDTA, and 10% Glycerol) containing protease inhibitors and phosphatase inhibitors, and then analyzed by immunoblotting with the indicated antibodies. The relevant antibody product numbers are as follows: USP15 (ab71713, Abcam, US), PhosphoSmad2 (Ser465/467)/Smad3 (Ser423/425) (#8828, CST, US), SMAD2 (#5339, CST, US), SMAD3 (#9523, CST, US), SMAD4 (46535, CST, US), SMAD7 (ab216428, Abcam, US), and GAPDH (# 5174S, CST, US) [45, 50].

### 2.14. Wound Healing Assay

Cells were seeded onto sterile 6-well plates and incubated at 37°C in complete RPMI 1640 medium to 100% confluence for the experiment and then changed to fresh serum-free media for another 12 h. Wounds were created in cells using a sterile 10.0 *μ*L pipet tip. The cells were washed twice with PBS, and the media in each well was replaced with 1 mL complete RPMI 1640. The compounds at specified concentrations were added to each well, and the cells were incubated at 37°C for 24 h under full conditions. Cell migration was observed and photomicrographed for quantitation and image analysis of each treatment [45, 46].

### 2.15. Coimmunoprecipitation Assay

CO-IP kit (ThermoScience) for CO-IP experiments. Four confluent plates of MDA-MB-231 breast cancer cells were selected and cell lysates were prepared in IP buffer (150 mM NaCl, 0.1% Triton X-ray, 100 mM Tris-HCl, 1 mM EDTA, and pH 7.4). After centrifugation at 14000 g for 20 min, the cell lysate supernatant was incubated with the corresponding antibody overnight. Cell lysates were incubated with A/G beads for 4 hours, then washed after centrifugation, and the eluates were resolved by SDS-PAGE and analyzed by western blotting. All CO-IP experiments were performed at 4°C [50, 51].

### 2.16. Statistics

The data are presented as the mean ± SD unless wise otherwise stated. Statistical tests were performed using GraphPad Prism, version 6.0 (GraphPad Software). For comparisons of 2 groups, an unpaired, 2-tailed Student's *t*-test was used. For comparison of multiple groups, 1-way ANOVA was used.

## 3. Results

### 3.1. Analysis of USP15 Expression in Different Tumors

**Figure 1:**
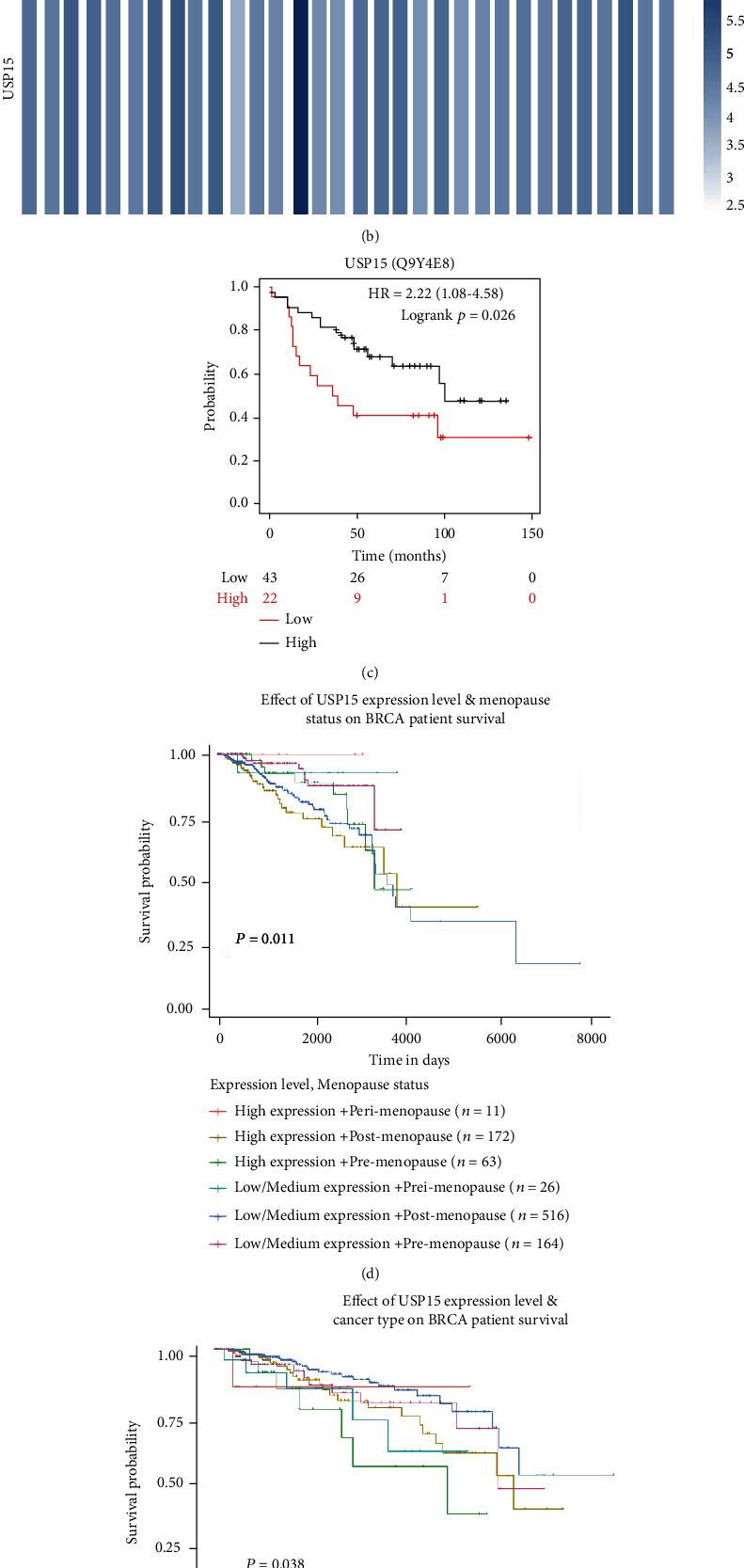
USP15's high expression of breast cancer has a poor prognosis. (a) Expression of USP15 in pancarcinoma, the data comes from UALCAN. (b) Expression of USP15 in pan-carcinoma, data from GEPIA. (c) USP15 expresses a correlation survival curve, data from Kaplan-Meier plotter. (d) Analysis of breast cancer survival curves associated with USP15 expression and pre- and postmenopausal, data from UALCAN. (e) Analysis of breast cancer survival curves related to the molecular classification of USP15 expression and breast cancer, analyzed from UALCAN.

Through the TCGA database, we found that USP15 was highly expressed in breast cancer, cholangiocarcinoma, esophageal cancer, head and neck squamous cell carcinoma, renal clear cell carcinoma, gastric adenocarcinoma, and acute myeloid leukemia (Figures 1(a) and 1(b)). Using Kaplan Meier survival curve analysis software, we subsequently found that high expression of USP15 led to worse survival in breast cancer (*p* = 0.026) (Figure 1(c)). Then, through the UALCAN and GEPIA database, we found that USP15 in breast cancer significantly reduced the survival rate of premenopausal women (*p* = 0.011) and triple-negative breast cancer patients (*p* = 0.038) (Figure 1(d) and 1(e)). Triple-negative breast cancer is the most challenging classification in the clinical treatment of breast cancer at present because triple-negative breast cancer has higher metastasis and invasion than other types of breast cancer, which often leads to poor treatment prognosis and increased high mortality. Therefore, we speculate that USP15 may be a key protein in the prediction of triple-negative breast cancer.

### 3.2. Analysis of Differential Expression of USP15 in Breast Cancer Protein Phosphorylation Plays an Important Role in Cell Signal Transduction

**Figure 2:**
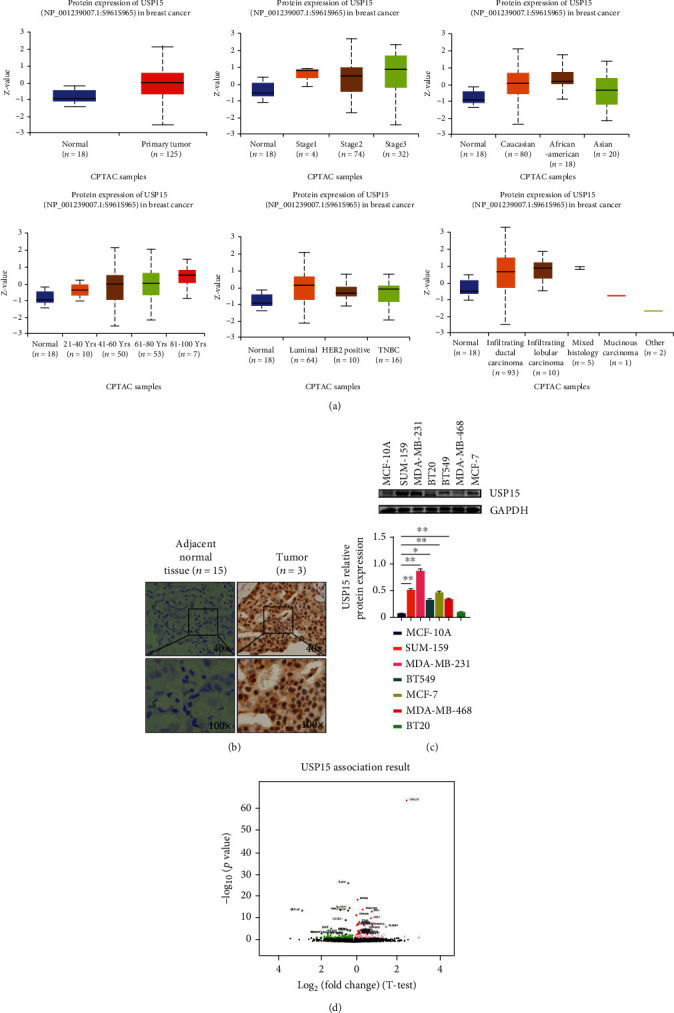
USP15 is highly expressed in breast cancer. (a) Expression of USP15 in different classification cases, data from UALCAN. (b) Through IHC experiments, the expression of USP15 in breast cancer tumor tissues and paracancerous normal tissues. (c) Through the western blotting experiment, the presentation and statistical analysis of USP15 in different breast cancer cell lines and human normal breast epithelial cells. (d) Screening of GENES Significantly Expressed in USP15 in BRCA (LinkedOmics) with Pearson Test.

Using the UALCAN database, we studied the expression of USP15 phosphorylation in breast cancer and normal tissues. The expression of phosphorylated USP15 in breast cancer tissues was higher than that in normal tissues. (*p* = 6.87e − 04) (Figure 2(a)), the phosphorylation expression of USP15 in different stages of breast cancer was also analyzed. The results showed that the expression of USP15 in different stages of breast cancer was stage 1 (*p* = 1.04e − 01), stage 2 (*p* = 4.05e − 03), and stage 3 (*p* = 4.11e − 03), respectively. The results showed that the expression of USP15 in stage 2 and stage 3 breast cancer was higher than in stage 1 breast cancer. At the same time, we also found that the expression of USP15 was different in the distribution of breast cancer in different races, including Caucasians (*p* = 5.12e − 04), African Americans (*p* = 1.51e − 03), and Asians (*p* = 5.12e − 04);*p* = 1.09e − 01). Since age influences tumor development and prognosis, we also performed a statistical analysis of breast cancer patients in different age groups. We found that the expression of USP15 was positively correlated with increasing age, with ages ranging from 21 to 40 years (4.28 e-02), 41-60 years old (4.92e-03), 61-80 years old (8.52e-04), and 81-100 years old (4.10e-03). Analyzing the results, we found that high expression of USP15 was mainly observed in breast cancer patients aged 41-60. To further analyze the influence of USP15 expression on breast cancer prognosis, we also performed a statistical analysis on breast cancer staging. Molecular typing results showed Luminal (*p* = 2.95e − 0.1), HER2 positive (*p* = 9.27e − 0.1), and TNBC (*p* = 6.68e − 01), and pathological typing results showed invasive ductal carcinoma (*p* = 3.98e − 01), invasive lobular carcinoma (*p* = 4.43e − 01), and mixed histology (*p* = 2.85e − 01)). The analysis results indicate that the expression level of USP15 is also closely related to the stage of breast cancer and that the expression of USP15 was higher in invasive breast cancer than in other types of breast cancer. Invasive breast cancer is one of the most common tumor types with a high potential for metastasis and a poor prognosis. Therefore, the above results suggest that USP15 plays an important role in the prognosis and survival of breast cancer patients. At the same time, we selected tumor tissue sections from 20 breast cancer patients from the pathology department of the General Hospital of Ningxia Medical University. By immunohistochemical analysis, we found that USP15 was a high expressed in tumor tissue sections from 3 of these patients. (Figure 2(b)). By reviewing relevant cases, we determined that the disease stage of these 3 patients was invasive breast cancer with metastases to vital organs. We then selected breast cancer cell lines: SUM159, MDA-MB-231, MDA-MB-468, BT549, and BT-20.MCF-7 and normal breast epithelial cell MCF -10A. USP15 expression was examined in vitro by Western blot analysis (Figure 2(c)). The results showed that the expression of USP15 in the triple-negative breast cancer cell lines was significantly higher than that of the MCF-7 ER-positive and PR-positive breast cancer cell lines. Among them, is triple-negative breast cancer cell line MDA-MB-231.SUM159, which has a strong invasive and metastasis ability, has a higher expression level, while normal breast epithelial cell MCF -10A has a lower expression level. Therefore, the above results further suggest that USP15 may be a key protein affecting the prognosis of breast cancer patients.

### 3.3. Expression of USP15-Related Genes in Breast Cancer to Study the Biological Role of USP15 in Breast Cancer

**Figure 3:**
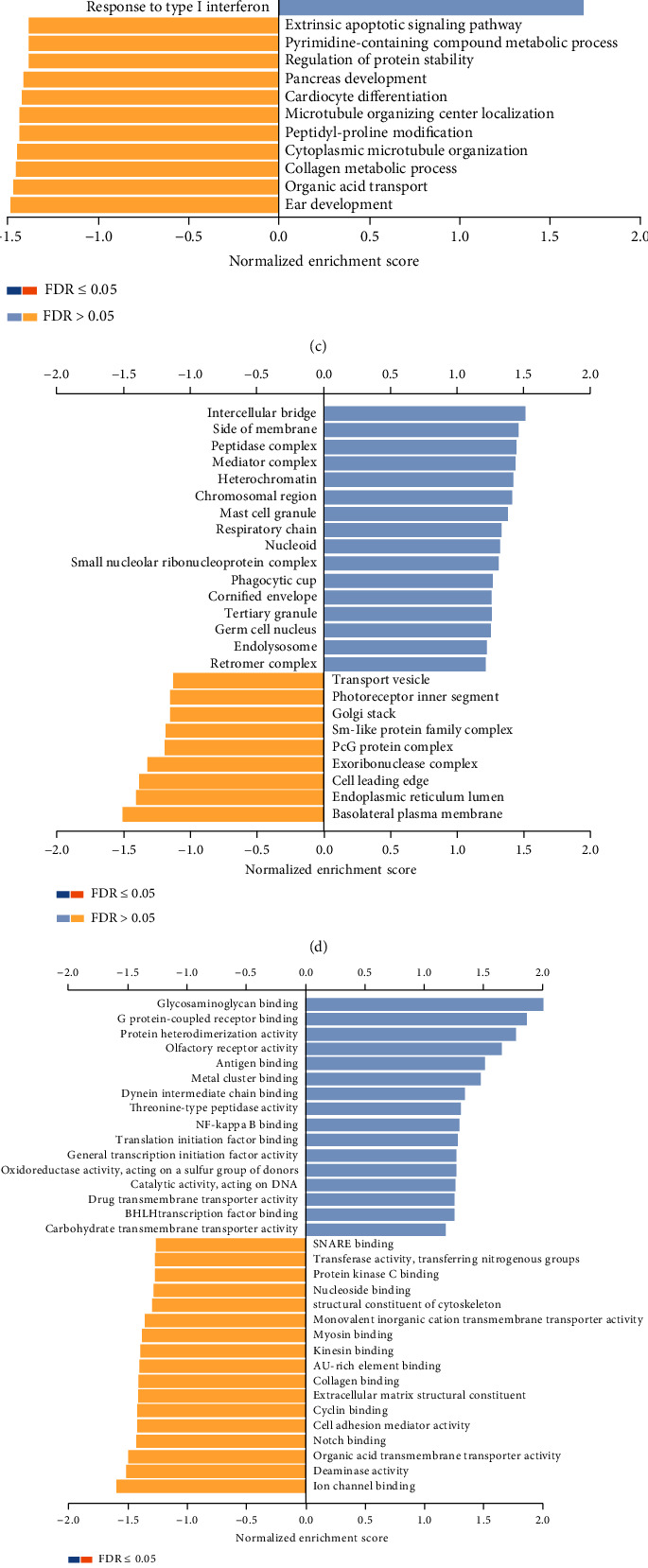
Differentially expressed genes associated with USP15 in breast cancer (LinkedOmics). (a, b) The first 50 significant genes related to USP15 in BRCA (c, d, e) GO enrichment analysis (Biological process; Cellular Component; Molecular Function) of USP15 in BRCA cohort (f) KEGG enrichments of USP15 in BRCA cohort.

LinkedOmics analyzed 509 BRCA patients with genes coexpressed by USP15, of which 3035 genes were positively correlated with USP15 and 2992 genes were negatively correlated with USP15 (false detection, FDR < 0.01) (Figure 2(d)). Showing the top 50 significant genes associated with USP15 in BRCA on the heat map (Figures 3(a) and 3(b)). We then performed GO accumulation analysis via the GESA database and found that the accumulation of differential upregulated USP15-related genes were primarily focused on angiogenesis, chemical synaptic transmission, postsynaptic, etc. in biological processes (Figure 3(c)). Among the cellular components, differential upregulated USP15-related genes were mainly enriched in microtubule-associated complexes, granular secretory membranes, etc. (Figure 3(d)). Meanwhile, in molecular functions, differential upregulated USP15-related genes were primarily enriched glycosaminoglycan binding, general transcription initiation factor activity, etc. (Figure 3(e)). We also performed KEGG pathway analysis. We found that genes coexpressed by USP15 were closely associated with cell adhesion molecule (CAM), natural killer cell-mediated cytotoxicity, and signaling pathways related to circulation (Figure 3(f)). The above results suggest that USP15 plays a vital role in cell adhesion and cell cycle regulation in breast cancer.

### 3.4. USP15-Associated Protein Analysis

**Figure 4:**
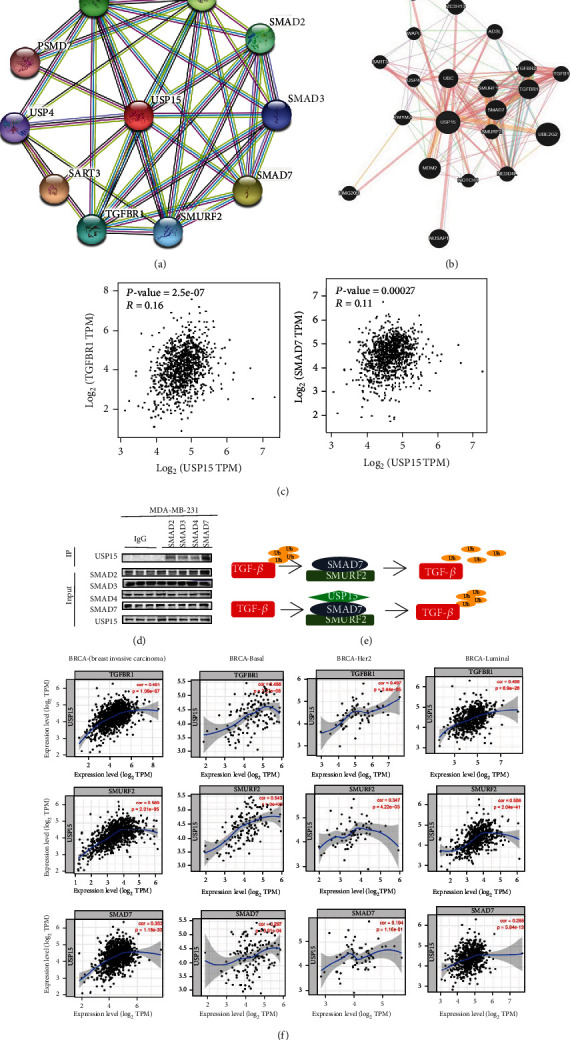
USP15 protein-protein interaction in BRCA. (a, b) Interaction network of USP15 gene derived from STRING and GeneMANIA. STRING and GeneMANIA show the interactions for genes linked with each other according to physical interaction, genetic interactions, coexpression, pathway, colocalization, and shared protein domain (c) USP15 correlation analysis; data from GEPIA (d) Immunoprecipitation experiments verify the interaction between USP15 and the Smad protein. (e) Schematic diagram of the structure in which USP15 functions (f) Correlation between USP15 and different proteins, data from TIMER.

Using the protein interaction network PPI from the STING database, we obtained many nodes: 12 and many edges: 51. Using the protein interaction results, we found that USP15 showed a higher correlation. Strong with TGF*β*RI compared to NPAS4 and CXCL13, where the combined value was 0.918. USP15 was also closely related to smad-related proteins and the combined score of USP15 and Smad7 was 0.976. (Figure 4(a)). We then validated our results using the GeneMANIA database (Figure 4(b)), which provided the same results as the STING search. We selected the protein interaction module in GEPIA analysis software and found the correlation coefficient between USP15 and TGF*β*RI (*R* = 0.16, *p* < 0.05). The correlation coefficient between USP15 and SMAD7 (*R* = 0.11, *p* < 0.05) (Figure 4(c)) The Smad protein is the first detected kinase substrate of the TGF-*β* receptor, so we hypothesize that USP15 in BRCA most likely plays a role in promoting tumor development and metastasis via the TGF-*β*/smad signaling pathway. To investigate how USP15 irregulates TGF-*β*/smad signaling in breast cancer, we first identified a strong protein interaction between USP15 and smad7, a kinase substrate of the TGF-*β* receptor, by experiments of coimmunoprecipitation. (Figure 4(d)). TGF*β* is a double-edged sword in the body as a transforming growth factor. It has been reported that when the body is in the tumor microenvironment, it mistakenly believes that TGF*β* is in a low expression state and TGF*β* is in a deregulated state, which subsequently destroys the body and stimulates production. Many USP15 combined with smad7, smurf2, and all three to deubiquitinate TGF*β* and increase TGF*β* signaling in vivo, thereby promoting tumor development (Figure 4(e)). To investigate the influence of USP15 on the prognosis of breast cancer patients, we decided to analyze the TIMER database and found that USP15 was associated with the expression of TGF*β*RI in the BRCA (Breast Invasive Carcinoma) type (Cor = 0.491, *p* = 1.08e − 67); smurf2 (Cor = 0.569), *p* = 2.01e − 95); and smad7 (Cor = 0.353, *p* = 1.18e − 33); in the basal BRCA type, USP15 was associated with the expression of TGF*β*RI (Cor = 0.456, *p* = 2.27e − 08); smurf2 (Cor = 0.543, *p* = 0e + 00); and smad7 (Cor = 0.297, *p* = 3.91e − 04); USP15 was BRCA-HER2 type with TGF*β*RI expression (Cor = 0.497, *p* = 2.44e − 05); smurf2 (Cor = 0.347, *p* = 4.22e − 03); and smad7 (Cor = 0.194, *p* = 1.16e − 01). Correlation of USP15 expression with TGF*β*RI in BRCA luminal type (Cor = 0.497, *p* = 2.44e − 05), smurf2 (Cor = 0.506, *p* = 2.04e − 01), and smad7 (Cor = 0.285, *p* = 5, 84e − 13) (Figure 4(f)). Based on the above database analysis results, we found that USP15 positively correlates with smad7, smurf2, and TGF*β*RI in different types of breast cancer. In summary, by combining smad7 and smurf2 in breast cancer, USP15 can form a trimeric complex, affect the TGF-*β*/smad signaling pathway, and promote tumor metastasis occurrence and its development.

### 3.5. The Role of USP15 Expression in Breast Cancer Growth, Metastasis, and Invasion

**Figure 5:**
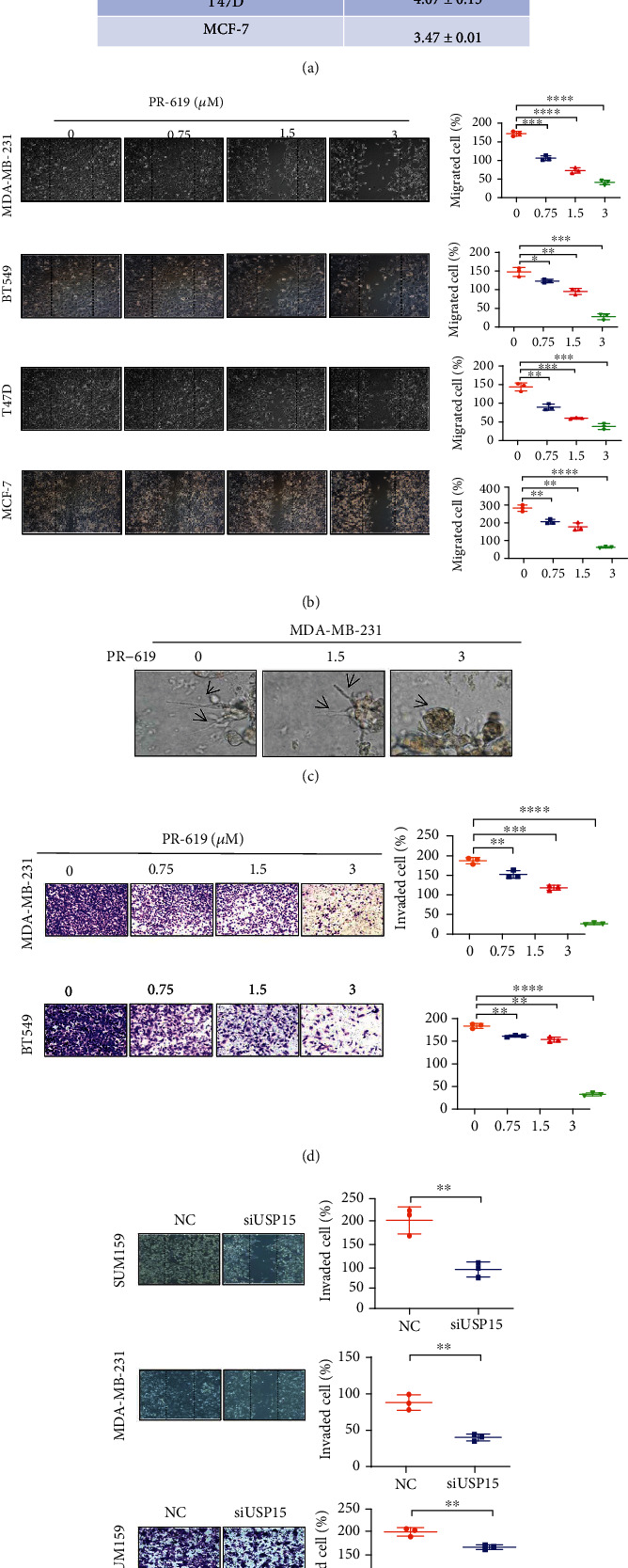
USP15 inhibits breast cancer cell metastasis and invasion (a) PR619 inhibits proliferation of breast cancer cells (b) PR619 inhibits breast cancer cell invasion (c) PR619 inhibits pseudopodia formation in breast cancer cells (d) PR619 inhibits breast cancer invasion (e) siUSP15 inhibits migration and invasion of breast cancer cells (f) Western blot analysis of key proteins inhibiting siUSP15 of TGF-*β*/smad signaling.

USP15 has not been extensively studied and no small molecule inhibitor against USP15 has emerged. Therefore, we selected a broad-spectrum inhibitor, PR619, which currently inhibits USP15, for consideration. First, we chose breast cancer cell lines MDA-MB-231, MDA-MB-468, BT549, MCF-7, and T47D for SRB cells from proliferation experiments, which showed that the half-inhibition rate of PR619 on MDA-MB-231 was 3.24 ± 0.22 *μ*M, the half-inhibition rate of MDA -MB-468 was 2.13 ± 0.08 *μ*M, BT549 half-inhibition rate was 6.79 ± 0.31 *μ*M, T47D MCF-7 half-inhibition rate was 4.07 ± 0.15 *μ*M, and the half-inhibition rate of MCF-7 was 3.47 ± 0.01 *μ*M. PR619 can inhibit the cell proliferation of various breast cancer cells in a concentration-dependent manner. Interestingly, we found that PR619 inhibited half of the proliferation of normal mammary epithelial cells at 13.73 ± 0.12 *μ*M, and PR619 interfered with GES-1(gastric epithelial cell) with a median inhibition rate of 1.91 ± 0.05 *μ*M, which suggests that PR619 could inhibit proliferation of breast cancer cells with less toxicity. (Figure 5(a)). To further verify the role of USP15 in metastasis and invasion of breast cancer cells, we then selected four breast cancer cells, MDA-MB-231, BT549, T47D, and MCF-7, for the assay of cell scraping and found that there was an increase in the concentration and dose of PR619 significantly inhibited the ability to migrate of breast cancer cells (Figure 5(b)) In tumor cells, tumor cell extended pseudopodia represent tumor cell motility. We used Matrigel 3D experiments to investigate further the effect of USP15 on the invasiveness of breast cancer cells. The results showed that tumor cell pseudopodia gradually decreased with increasing concentration and dose, representing tumor motility's attenuation (Figure 5(c)). At the same time, we selected 2 triple negative breast cancer cell lines and then checked them by the transwell Invasion Assay. The results showed that PR619 could significantly inhibit the invasion of breast cancer cells. (Figure 5(d)). Since PR619 is a broad-spectrum inhibitor and cannot be well represented, we selected siUSP15 and performed verification by cell scraping and ventricular invasion assay on two cell lines of MDA-MB-231 and SUM159. Through the experimental results, we found that the siUSP15 group can significantly inhibit the invasion and metastasis of breast cancer in the phenotype compared with the NC group, so we can assume that the USP15 plays a role in breast cancer tumor cell invasion and metastasis (Figure 5(e)). The role of USP15 in inhibiting breast cancer growth, metastasis, and the attack has been further investigated. Therefore, we downregulated the expression of USP15 in two breast cancer cell lines, MDA-MB-231 and SUM159, and performed a Western blot analysis of the expression of key proteins in the signaling pathway. The results showed that the siUSP15 group significantly reduced the protein expression of the key protein *p*-smad2/3 in the signaling pathway compared to the NC group (Figure 5(f)). From the above experimental results, we can assume that USP15 plays a key role in breast cancer initiation, metastasis, and invasion.

### 3.6. The Effect of USP15 on Immune Function in Breast Cancer Patients

**Figure 6:**
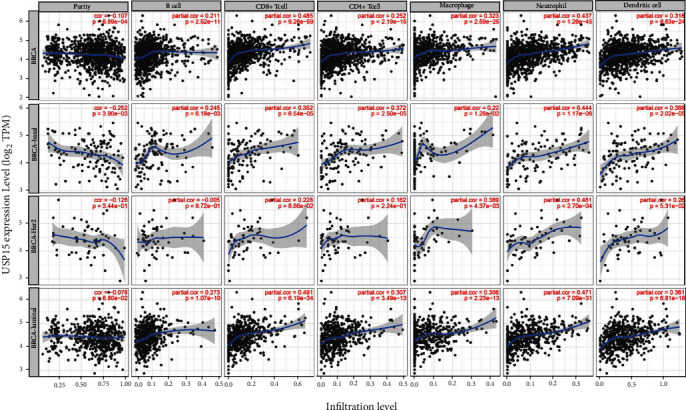
Correlation of USP15 with immune cell infiltration (TIMER) in different breast cancer types, data from TIMER.

>USP15 is involved in influencing breast cancer metastasis and invasion. To further investigate the impact of USP15 on the prognosis of breast cancer patients, we used the TIMER database to examine the effect of USP15 on immune infiltration in different types of breast cancer. In BRCA types, USP15 expression was associated with B cells (COR = 0.211, *p* = 2.52e − 11), CD8+ T cells (COR = 0.485, *p* = 9.26e − 59), CD4+ T cells (COR = 0.252, *p* = 2.19e − 15), Macrophage (COR = 0.323, *p* = 2.59e − 25), Neutrophil (COR = 0.437, *p* = 1.26e − 45), and Dendritic cell (COR = 0.318, *p* = 8.63e − 24) were positively correlated; in BRCA-Basal types, USP15 expression was associated with B cells (COR = 0.245, *p* = 6.18e − 03), CD8+ T cells (COR = 0.352, *p* = 6.54e − 05), CD4+ T cells (COR = 0.372, *p* = 2.50e − 05), Macrophage (COR = 0.22, *p* = 1.28e − 02), Neutrophil (COR = 0.444, *p* = 1.17 e − 06), and Dendritic cell (COR = 0.388, *p* = 2.02e − 05) were positively correlated; in BRCA-Her2 type, USP15 expression was negatively correlated with B cells (COR = −0.005, *p* = 9.72e − 01), negatively correlated with CD8+ T cells (COR = 0.228, *p* = 8.86e − 02), CD4 + T cells (COR = 0.162, *p* = 2.24e − 01), Macrophage (COR = 0.369, *p* = 4.37e − 03), and Neutrophage (COR = 0.369, *p* = 4.37e − 03)),Neutrophil (COR = 0.461, *p* = 2.70e − 04) and Dendritic cell (COR = 0.26, *p* = 5.31e − 02) were positively correlated; in BRCA-Luminal type In BRCA-Luminal type, USP15 expression was positively correlated with B cells (COR = 0.273, *p* = 1.07e − 10), with CD8+ T cells (COR = 0.491, *p* = 6.19e − 34), CD4+ T cells (COR = 0.307, *p* = 3.49e − 13), Macrophage (COR = 0.308, *p* = 2.23e − 13), Neutrophil (COR = 0.471, *p* = 7.09e − 31), and Dendritic cell (COR = 0.361, *p* = 6.81e − 18) were positively correlated (Figure 6(a)). Next, we performed a meta-analysis of factors related to survival and found that in addition to USP15 expression affecting prognosis in breast cancer patients, age and stage of tumor also had a significant association with prognosis in patients with breast cancer (Table 1).

## 4. Discussion

The current clinical treatment of breast cancer mainly aims to eliminate cancer cells as the primary goal. However, cancer cells can often evade the ability of clinical chemotherapeutic agents to induce death programs, resulting in clinically detectable recurrence or metastasis. Breast cancer metastasis is currently the leading cause of the vast majority of cancer deaths and a significant barrier to breast cancer treatment. This requires the development of more specific approaches to tumor metastases; we found that a USP15 target has a significant inhibitory effect on breast cancer metastasis in vitro.

USP15 is the 16th protease in the USP family, it is composed of 952 small amino acid molecules and the structure contains a conserved catalytic hydrolysis domain composed of cysteine (Cys) and histidine (His) [52]. USP15 has been shown to induce angiogenesis and promote tumor invasion and is a key protein involved in tumorigenesis. [53]. However, USP15 has not been widely studied, so this study proposes that high USP15 expression is significantly associated with poor prognosis in breast cancer patients and reveals a potential regulatory network [20].

In this study, we found that USP15 was highly expressed in breast cancer by searching the TCGA databases [32, 54]. Then by searching the bioinformatics database UALCAN, GEPAI, Kaplan-Meier we. found that high expression of USP15 significantly affects the prognosis of premenopausal breast cancer patients aged 40-60 years. To verify the effect of USP15 on the prognosis of breast cancer patients, tumor tissues from breast cancer patients were first selected for immunohistochemical experiments. The results showed that USP15 was highly expressed and then triple expressed in breast cancer patients with metastases. Negative breast cancer cell lines were selected in vitro. Western blot experiments then verified the expression of USP15. We found that the expression of USP15 in the two cell lines with the strongest metastatic and invasive abilities, MDA-MB-231 and SUM159; this result suggests that USP15 may affect breast cancer prognosis by altering the ability of tumor metastasis and invasion [55]. In addition, GO enrichment analysis and KEGG pathway analysis via the GESA database were performed on USP15. The study showed the biological function and accumulation analysis of the USP15 KEGG pathway in breast cancer patients primarily related to cell adhesion and cell metastasis. Molecular inhibitor PR619 was validated in vitro by cell scraping test, chamber invasion test, and 3D Matrigel test, and USP15 was shown to affect metastasis and invasion of patients with breast cancer. Meanwhile, to further explore the mechanism of USP15, we first obtained the associated proteins and associated functional networks of USP15 in breast cancer patients by searching bioinformatics databases such as STRING and GeneMANIA to examine associated signaling pathways involved in breast cancer USP15. Importantly, we found that there is also a relationship between USP15 expression in breast cancer patients and immunity expression in the data mining process. This result indicates that immune processes may also regulate the effect of USP15 in breast cancer patients. USP15 expression affects the prognosis of breast cancer patients and is highly expressed in breast cancer cells with strong metastatic and invasive abilities, suggesting that USP15 may affect tumor metastasis and invasion. In the market which targets USP15, the broad-spectrum inhibitor PR619, which has an inhibitory effect on USP15, was selected in this study for preliminary investigation. The selected siUSP15 was verified Based on the experimental results, we found that low expression of USP15 may decrease the ability of breast cancer cells to metastasize and invade. Overexpressed USP15 was not chosen for a rescue experiment. It has been reported in the literature that the USP15 inhibits tumors by reducing the deubiquitination of TGFBR1. This point has not been verified in this article, and the mechanism of action of USP15 will be studied in the future.

In conclusion, our results suggest that breast cancer metastasis can be blocked by knocking down USP15. The results of this work may reflect the role of USP15 in tumor metastasis, providing an opportunity to assess the clinical relevance of USP15 for metastasis. Our results also offer the opportunity to develop drugs targeting USP15; USP15 may be an attractive predictor of breast cancer prognosis and merits further study in treating breast cancer.

## Figures and Tables

**Table 1:** BRCA and USP15 meta-analysis with related factors such as sex, age, tumor typing, immune cell type, and data from TIMER.

	Coef	HR	95%CI_1	95XCI_U	p.value	Sig
Age	0.034	1.035	1.020	1.049	0.000	^∗∗∗^
Gendermale	-0.490	0.613	0.084	4.459	0.628	
raceBlack	-e.064	0.938	0.278	3.169	0.918	
raceWhite	-9.533	0.587	0.182	1.894	0.372	
stage2	8.580	1.786	0.990	3.222	0.054	^∗^
stage3	1.288	3.625	1.947	6.750	0.000	^∗∗∗^
stage4	2.688	14.700	6.677	32.367	0.000	^∗∗∗^
B_cell	-6.684	0.505	0.006	41.251	0.761	
CD8_Tcell	-1.733	0.177	0.015	2.144	0.174	
C04_Tcell	0.197	1.218	0.030	50.000	0.917	
Macrophage	2.439	11.466	0.849	154.784	0.066	^∗^
Neutrophil	1.4S6	4.420	0.019	1017.417	0.592	
Dendritic	-8.494	0.610	0.081	4.597	0.632	
USP15	0.380	1.462	1.001	2.135	0.049	^∗^

Rsquare-0.085 (max possible = 7.96e − 01). Likelihood ratio test *p* = 3.62e-ll. Wald test *p* = 2.65e − 13. Score (logrank) test *p* > = 1.79e − 18.

## Data Availability

The data used to support the findings of this study can be obtained through the corresponding author upon reasonable request.
